# Genetic Structure of the Spanish Population

**DOI:** 10.1186/1471-2164-11-326

**Published:** 2010-05-25

**Authors:** Javier Gayán, José J Galan, Antonio González-Pérez, María Eugenia Sáez, María Teresa Martínez-Larrad, Carina Zabena, M Carmen Rivero, Ana Salinas, Reposo Ramírez-Lorca, Francisco J Morón, Jose Luis Royo, Concha Moreno-Rey, Juan Velasco, José M Carrasco, Eva Molero, Carolina Ochoa, María Dolores Ochoa, Marta Gutiérrez, Mercedes Reina, Rocío Pascual, Alejandro Romo-Astorga, Juan Luis Susillo-González, Enrique Vázquez, Luis M Real, Agustín Ruiz, Manuel Serrano-Ríos

**Affiliations:** 1 Department of Structural Genomics, Neocodex, Sevilla, Spain; 2 CIBER de Diabetes y Enfermedades Metabólicas Asociadas (CIBERDEM), Department of Internal Medicine II, Hospital Clínico San Carlos, Madrid, Spain

## Abstract

**Background:**

Genetic admixture is a common caveat for genetic association analysis. Therefore, it is important to characterize the genetic structure of the population under study to control for this kind of potential bias.

**Results:**

In this study we have sampled over 800 unrelated individuals from the population of Spain, and have genotyped them with a genome-wide coverage. We have carried out linkage disequilibrium, haplotype, population structure and copy-number variation (CNV) analyses, and have compared these estimates of the Spanish population with existing data from similar efforts.

**Conclusions:**

In general, the Spanish population is similar to the Western and Northern Europeans, but has a more diverse haplotypic structure. Moreover, the Spanish population is also largely homogeneous within itself, although patterns of micro-structure may be able to predict locations of origin from distant regions. Finally, we also present the first characterization of a CNV map of the Spanish population. These results and original data are made available to the scientific community.

## Background

The large genotyping studies in the last decade have revolutionize genetic studies. Our current ability to characterize the human genome is unprecedented [1-3], and is contributing to improve our understanding of the genetic etiology of common diseases.

Genetic admixture is one of the caveats for genetic association studies [4], and has fostered the comparative study of the genetic structure of different human populations. A large number of studies are underway to identify the similarities and differences among existing human populations [2,3]. These studies started comparing the general human populations such as Africans, Asians and Europeans, but have recently focused on the more specific subgroups within them [5-8]. It seems that, as genetically similar as humans are, we can now tune the genetic "microscope" so that subtle genetic differences among related subpopulations can be detected [9], even among regions within a country [10,11].

The Neocodex Biobank and Genome Research Consortium is planning a number of genome-wide association studies (GWAS) in several complex phenotypes. Our basic and general strategy will consist in the systematic comparison of a well-characterized population-based control dataset against a number of datasets of complex phenotypes, such as metabolic syndrome, osteoporosis, Alzheimer's disease, colorectal cancer or multiple sclerosis. Therefore, it is markedly important to select individuals representative of the genetic diversity co-existent in Spain and to make an in-depth genomic characterization of these control individuals that will serve as a reference panel for future GWAS studies.

As an initial step of our investigation, we decided to characterize the genetic structure of the Spanish population using high density SNP arrays. This study lays an essential base for future GWAS, by identifying potential sources of bias that may affect experimental results and that could increase the noise and false positive rate of GWAS in our population. Furthermore, this work begins the characterization of common copy number variants (CNVs) in our population that might interfere with association studies in discrete regions of the genome or that may be related to the phenotypes by itself.

In this study, we have analyzed linkage disequilibrium (LD) patterns and haplotype blocks in the population of Spain, and compared them to Western and Northern Europeans. We have also estimated population stratification and substructure, and have identified CNVs in this sample of the Spanish population.

## Results

801 Spanish individuals were genotyped with the Affymetrix Nsp I 250 K chip, from which 166,588 SNPs passed the quality control filters, and were used in the LD, haplotypic and structure analyses described below. In addition, genotype data from the HapMap project were used for comparison purposes: we selected the genotypes from the same chip for 60 unrelated CEU individuals. Moreover, subsets of HapMap individuals with European, African, and Asian ancestry were employed in the principal components analysis.

### Allele Frequencies

**Figure 1:**
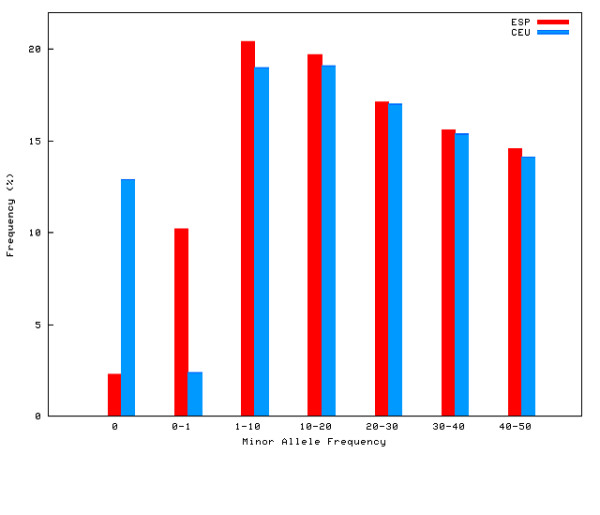
**Allele Frequencies**. Minor allele frequency distribution in the Spanish (ESP, in red) and CEU Hapmap (in blue) samples. Results show that the frequency distribution of common SNPs (MAF = 10 - 50%) are very similar in the two populations.

The average minor allele frequency (MAF) across all autosomal SNPs (mean = 0.203, median = 0.186) was almost identical to that of the CEU HapMap sample (mean = 0.201, median = 0.183). The distribution of MAF is not uniform. 2.3% (N = 5978) of the SNPs were monomorphic, 10.2% (N = 26253) were rare alleles (MAF = 0-1%), and 20.4% (N = 52367) were low-frequency alleles (MAF = 1-10%). The distribution of the remaining, common SNPs (MAF = 10-50%) was more uniform, although frequency declines as MAF increases. Figure 1 compares the MAF distributions between the Spanish (ESP) and CEU Hapmap samples, showing that the frequency distribution of common SNPs (MAF = 10-50%) are very similar.

### LD and haplotypic structure

**Figure 2:**
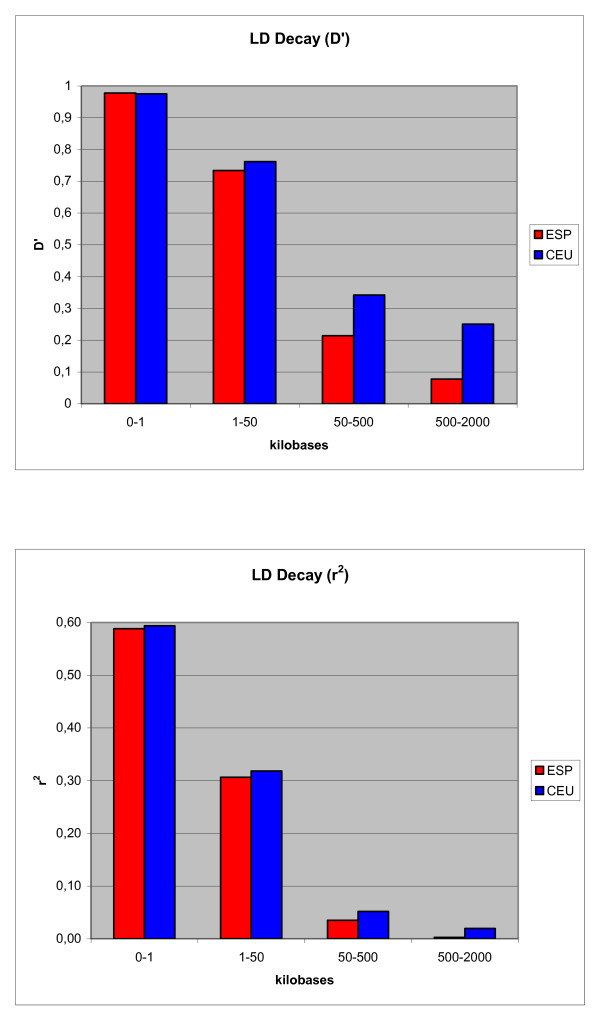
**LD decay, represented as D' and r2 averages for several SNP-distance ranges, in the Spanish (ESP, in red) and CEU Hapmap (in blue) samples**. It is shown that, in general, LD decreases as genetic distance increases.

**Figure 3:**
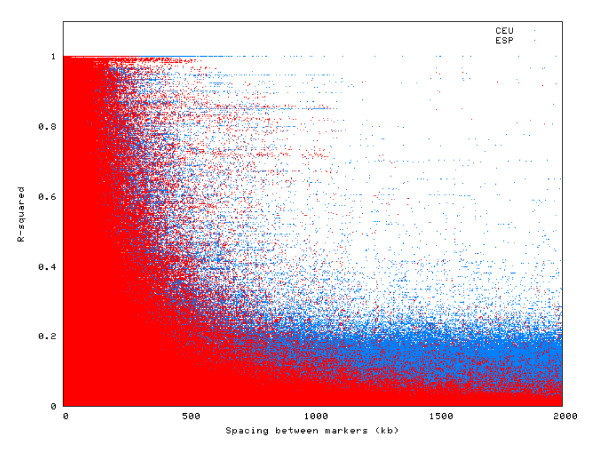
**Two-locus LD values (r2) for all genomewide pairs of SNPs less than 2 megabases apart, in the Spanish (ESP, in red) and CEU Hapmap (in blue) samples**. This figure shows graphically how LD decreases exponentially with genetic distance, and it also displays the large variability around this general trend.

It is well known that LD decreases exponentially with genetic distance, and this pattern is confirmed in the Spanish population analyzed in this study. Figure 2 represents visually this LD decay. Specifically, for SNPs up to 1 kb apart, LD is large (average D' = 0.98, average r2 = 0.59). For SNPs up to 50 kb apart, the average D' is 0.73 (average r2 = 0.31). For markers between 50 and 500 kb apart, the average D' decreases to 0.21 (r2 = 0.03). Moreover, for markers 500 kb-2 Mb apart, the average D' is only 0.08 (r2 = 0.002). This general pattern shows, nonetheless, large variability (Figure 3). For example, maximal LD (D' or r2 = 1.0) can be exceptionally observed across pairs of markers several Mb away. But in general, high LD (D' > 0.95 or r2 > 0.8) is very rare over distances of 500 kb or above. On the other side of the scale, low LD (D' < 0.20 or r2 < 0.10) can be observed in markers less than 1 kb apart. These values are similar to those obtained from the CEU HapMap Nsp I 250K dataset, but LD is greater in the CEU sample, especially for markers farther apart (D' values for the above distances are 0.98, 0.76, 0.34, and 0.25) (Figures 2 and 3). This is possibly due to the smaller size of the CEU sample (N = 60), where less chromosomes are represented, and thus less chances exist of a recombination event breaking long-range LD.

Beyond pair-wise LD patterns, haplotype blocks give a more global description of LD structure. In this sample that represents the population of Spain, we have estimated 33,037 haplotype blocks in the 22 autosomal chromosomes. A list of haplotype blocks, including haplotype frequencies, LD between adjacent haplotypes, and multiallelic LD between adjacent blocks, is included as Additional File 1. Each block covers 3.97 SNPs on average, ranging from small blocks of only 2 SNPs to some very large blocks of as much as 64 SNPs. This largest block is located in chromosome 17q21.31:41,097,235-42,177,829, between rs17760577 and rs199535. This 17q21.31 region is a gene-rich region (including CRHR1 and MAPT) exhibiting large LD blocks (approximately 623 kb) in the HapMap Phase II dataset in all populations studied (YRI,CEU, and JPT+CHB), and with an interesting evolutionary story involving a large inversion [12].

The number of blocks (Range = 323-2834) and block size (Range = 14-28kb) per chromosome, summarized in Table 1, is quite variable among the chromosomes. The block structure in the Spanish population is in general similar to that of the CEU HapMap sample, but with important differences. The Spanish sample exhibits more but smaller blocks than the CEU sample. This finding, again, may be a consequence of the difference in sample size. The more chromosomes represented in the Spanish sample may reveal recombination events that break down blocks, while these same blocks in the CEU sample may extend further distances.

**Table 1:** Block Structure.

Chromosome	# Blocks		Total block size (bp)		Mean block size (bp)		Block coverage (%)	
	ESP	CEU	ESP	CEU	ESP	CEU	ESP	CEU
1p	1249	948	32.737.822	27.342.410	26.211,2	28.842,2	27,26	22,76
1q	1252	944	30.080.036	26.585.943	24.025,6	28.163,1	29,07	25,69
2p	1225	941	24.637.280	22.502.333	20.112,1	23.913,2	27,75	25,34
2q	1609	1241	46.205.456	39.289.507	28.716,9	31.659,6	31,28	26,60
3p	1184	848	27.197.150	24.057.572	22.970,6	28.369,8	30,31	26,81
3q	1203	963	32.903.956	30.465.304	27.351,6	31.635,8	31,55	29,21
4p	637	492	12.930.719	11.278.505	20.299,4	22.923,8	26,56	23,16
4q	1754	1349	43.830.154	39.314.219	24.988,7	29.143,2	31,59	28,33
5p	604	453	14.182.170	12.154.003	23.480,4	26.830,0	30,61	26,23
5q	1611	1218	43.221.714	38.561.489	26.829,1	31.659,7	32,99	29,43
6p	822	627	18.447.817	15.585.108	22.442,6	24.856,6	31,42	26,54
6q	1428	1084	40.564.830	35.577.295	28.406,7	32.820,4	37,34	32,75
7	1899	1407	43.511.795	38.559.814	22.913,0	27.405,7	27,45	24,33
8	1936	1453	43.620.677	39.732.026	22.531,3	27.344,8	30,66	27,93
9	1577	1162	30.454.999	27.177.922	19.312,0	23.388,9	28,06	25,05
10	1804	1348	39.944.618	33.757.395	22.142,2	25.042,6	30,17	25,50
11	1634	1263	43.842.615	37.543.778	26.831,5	29.725,9	33,45	28,65
12	1595	1230	40.870.499	35.590.775	25.624,1	28.935,6	31,26	27,22
13	1404	1125	32.055.904	27.707.093	22.831,8	24.628,5	33,40	28,87
14	1079	803	24.587.012	21.628.792	22.786,9	26.935,0	28,31	24,90
15	946	691	18.423.385	16.851.087	19.475,0	24.386,5	22,65	20,72
16	952	721	13.905.324	12.405.433	14.606,4	17.205,9	17,79	15,87
17	647	456	16.392.095	14.733.568	25.335,5	32.310,5	20,86	18,75
18	1062	787	20.833.668	18.414.659	19.617,4	23.398,6	28,24	24,96
19	323	251	6.660.803	4.956.302	20.621,7	19.746,2	12,12	9,02
20	745	594	14.402.439	12.942.356	19.332,1	21.788,5	24,32	21,85
21	526	401	7.978.550	7.073.615	15.168,3	17.639,9	23,95	21,23
22	330	232	5.880.322	5.124.751	17.819,2	22.089,4	17,29	15,07

The number of blocks, total and average block size, and proportion of chromosome covered by blocks in the Spanish (ESP) and CEU samples.

Nonetheless, it is noteworthy that a larger portion of the genome is covered by blocks in the Spanish sample (28%), than in the CEU sample (24%). Again, the percentage of chromosomes covered by blocks is quite variable across the chromosomes, ranging between 12.12% for chromosome 19 and 37.34% for chromosome 6q.

### Population Stratification

Population stratification was analyzed with the STRUCTURE and EIGENSOFT softwares. Two sets of SNPs were analyzed: Subset A consists of 2,050 unlinked SNPs, while subset B includes 102,850 SNPs selected under less stringent criteria for marker relatedness.

**Figure 4:**
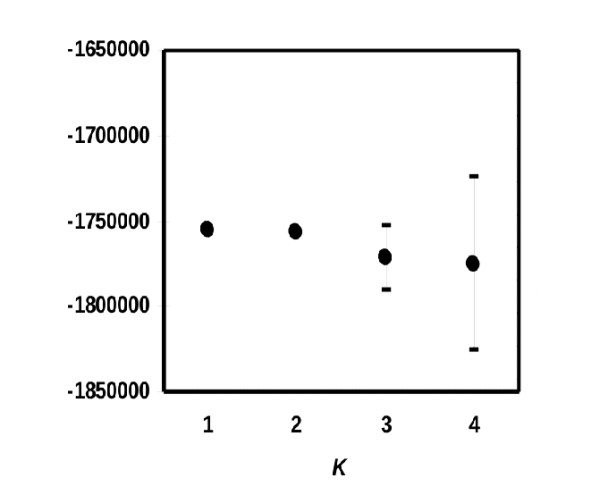
**Structure Analysis of the Spanish sample**. Mean L(K) +/- Standard Deviation (in brackets) over ten runs for each value of K (1, 2, 3 and 4). The best mean likelihoods were obtained assuming one single population (K = 1) and two populations (K = 2).

Figure 4 shows the mean L(K) and standard deviations for each value of k, obtained with the STRUCTURE analysis of marker subset A. The best mean likelihoods were obtained assuming one single population (k = 1) and two populations (k = 2). The likelihood decreased thereafter. According to simulations, the likelihood tends to increase until the true K is reached, and then levels off (often still slightly increasing) and the variance between runs increases [13]. Our data are therefore fully compatible with one true underlying population according to this analysis.

**Figure 5:**
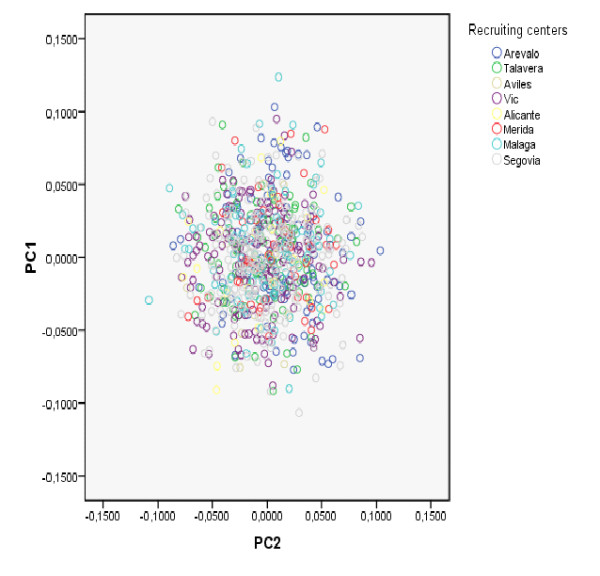
**PC results (marker subset A) for Spanish sample**. Distribution of Spanish individuals according to the top two Principal Components, using SNP marker subset A which includes only 2,050 unlinked SNPs (two-locus r^2 ^< 0.011).

Analyzing the same subset of markers (subset A) with EIGENSOFT resulted in similar conclusions. Figure 5 shows the distribution of the 801 individuals according to the top 2 principal components. Individuals from each recruiting center are coded by a different color. Although recruiting centers are widely distributed across Spain, the distribution of individuals from different geographic regions clearly overlap in the graph, suggesting there are no major differences between them. The Tracy-Wisdom test was borderline significant for the first PC (p = 0.047) and non-significant for the second (p = 0.331).

**Figure 6:**
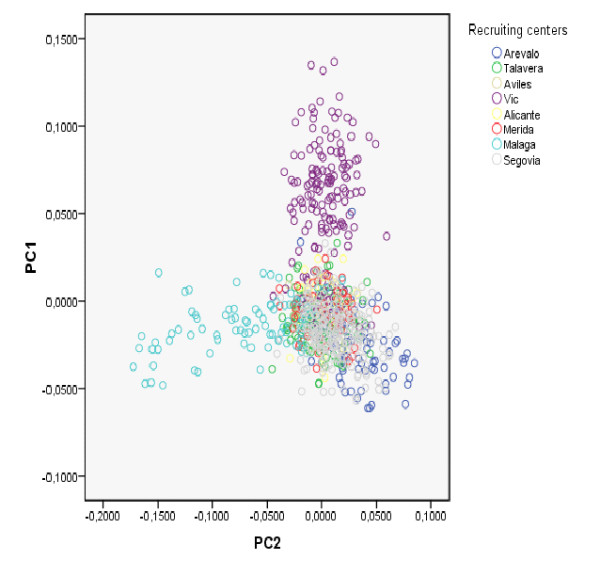
**PC results (marker subset B) for Spanish sample**. Scatter plot of the top two Principal Components from the analysis of the Spanish sample, using SNP marker subset B which includes 102,850 SNPs (two-locus r^2 ^< 0.8; Long-range LD regions excluded from analysis).

**Figure 7:**
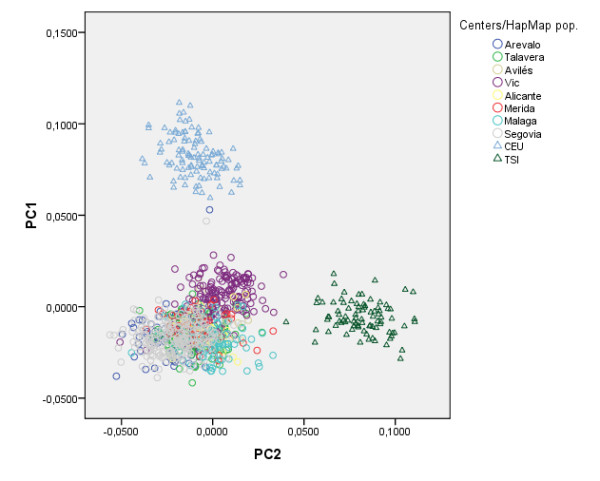
**PC results (marker subset B) for European samples**. Scatter plot of the top two Principal Components from the analysis of the Spanish sample and two European HapMap samples, using SNP marker subset B. HapMap sample acronyms stand for: CEU: Utah residents with ancestry from northern and western Europe; TSI: Toscani in Italy.

**Figure 8:**
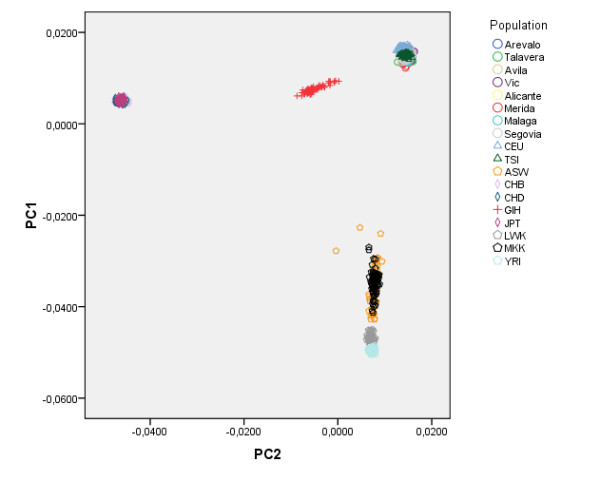
**PC results (marker subset B) for Worldwide samples**. Scatter plot of the top two Principal Components from the analysis of the Spanish sample and multiple HapMap samples, using SNP marker subset B. HapMap sample acronyms stand for: European (CEU: Utah residents with ancestry from northern and western Europe; and TSI: Toscani in Italy), African (ASW: African ancestry from Southwest USA; LWK: Luhya in Webuye, Kenya; MKK: Maasai in Kinyawa, Kenya; YRI: Yoruba in Ibadan, Nigeria) and Asian ancestry (CHB: Han Chinese in Beijing, China; CHD: Chinese in Metropolitan Denver, Colorado; GIH: Gujarati Indians in Houston, Texas; JPT Japanese in Tokyo, Japan).

**Figure 9:**
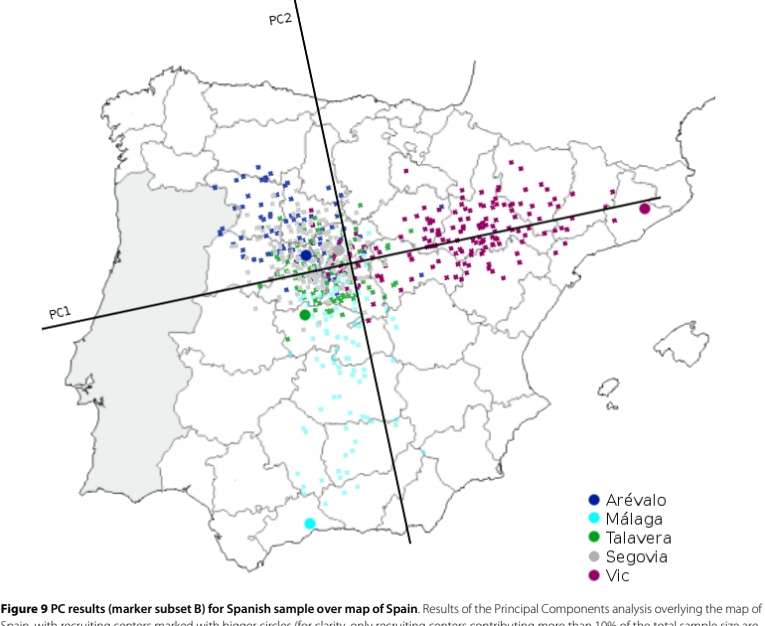
**PC results (marker subset B) for Spanish sample over map of Spain**. Results of the Principal Components analysis overlying the map of Spain, with recruiting centers marked with bigger circles (for clarity, only recruiting centers contributing more than 10% of the total sample size are shown).

Running PC analysis on marker subset B offers quite a different picture. As shown in Figure 6 the distribution of individuals from the two most geographically distant recruiting centers (Málaga and Vic) is quite different in this analysis. The province of Málaga is located in the south of Spain while Vic (Barcelona) is north-east of Spain, and they are located 1000 Km apart. We also observe a less striking gradient represented by individuals from Arévalo (Ávila), but not from the small number of individuals (N = 18) from Avilés (Asturias) who geographically are located farther apart in this direction. All other centers are located both geographically and on this plot somewhere in between Málaga and Vic. As expected, when European HapMap datasets are included (CEU with Northern and Western European ancestry individuals and TSI samples from Toscani in Italy), Spanish samples from different centers appear closer together in the graph. However the south to north-east axis (i.e. Málaga to Vic) observed in Figure 6 is still appreciated in this analysis and seems to correspond to a more general continent-wide south-west to north-east axis (Figure 7). Figure 8 shows the result of the PC Analysis when Hap Map datasets with African and Asian ancestry are also included. Returning to the analysis of the Spanish population, and in order to evaluate graphically the resemblance of this genetic distribution to the geographic distribution of the recruiting centers, the PC axes have been flipped horizontally, rotated 78 degrees clockwise, and superimposed over a map of Spain in which the location of the recruiting centers have been marked with large colored dots (Figure 9). To aid visualization, only individuals from recruiting centers contributing more than 10% of the total sample size are shown, according to the same two PCs from Figure 6. Tracy-Wisdom test were extremely significant for the top 2 PCs (p < 10^-78^).

### CNV

A total of 11,743 CNVs were identified in our sample set (14.70 CNVs per individual on average). With the aim of avoiding as much false positive results as possible, we will only consider here those 623 CNVs present in, at least, three individuals (Additional File 2).

Overall, those CNVs span 70.64 Mb of human autosomal genome and chromosome X. Mean (SD) and median sizes for those variants are 194.02 (205.26) Kb and 150.70 Kb, respectively, with a range of 10.15 Kb to 2,475.57 Kb. Population frequency ranges from 0.37% to 44.94%, but only 214 CNVs have frequencies above 1%. Most of the CNVs detected are copy number gains (47.51%), followed by copy number losses (26.64%), and copy number gains and losses (25.84%). We did not detect any difference in mean population frequencies among copy number states. However, copy number losses are lower in size than copy number gains (147.33 Kb versus 203.82 Kb; Mann-Whitney U test p < 0.01).

Some of the CNVs identified in this study (83.31%) overlap fully or partially with previously described structural variants. The mean (SD) and median nucleotide coverage of identified CNVs by previous CNVs (those included in DGV) are 60% (44%) and 87%, respectively. There is a positive correlation among the population frequency of the CNVs and their base pair coverage by previously detected structural variants (Spearman's rho = 0.24; p < 0.01). We detected 104 new CNVs (16.69%) and none of them were above 7.37% of population frequency.

It has been proposed that genomic regions flanked by segmental duplications (SD) (i.e. genomic stretches from 1 to 400 Kb in length with > 0.90 similarity) are susceptible to structural variations by nonallelic homologous recombination (NHR) [14]. To investigate whether NHR could account for some of the CNVs in our sample set, we calculated the percentage of CNVs included in genomic rearrangement hotspots. These regions were defined as DNA stretches of 50 Kb to 10 Mb in length, flanked by intrachromosomal SD > 10 Kb in size, in a similar way than Sharp et al. (2005). Indeed we found that 217 CNVs (34.83%) are included in rearrangement hotspots. Interestingly, we observed that those CNVs are statistically more frequent than CNVs located out of rearrangement hotspots (mean frequencies 2.47% and 1.34%, respectively; Mann-Whitney U test p < 0.01). In addition, the percentage of copy number states are statistically different among those two groups of CNVs, since those within rearrangement hotspots present a higher percentage of copy number gains and losses (both) when compared to CNVs out of these regions (42.86% and 16.75%, respectively; Pearson X^2 ^= 50.37; p < 0.01).

To analyse the impact of CNVs on genomic functional elements, we created a gene interval map comprising 22,738 known genes (refseqs) at autosomes and chromosome X. 553 CNVs (88.76%) overlap at least one gene interval. It has been suggested that deletions are biased away from genes [15]. We observed that the median number of genes is lower in copy number losses (mean = 3.31; SD = 3.05) when compared to copy number gains (mean = 4.14; SD = 5.01) but this difference does not reach statistical significance in our sample set. We identified 154 CNVs overlapping 125 loci included at the morbidmap list ftp://ftp.ncbi.nlm.nih.gov/repository/OMIM/.

The impact of CNVs on genomic surveys was also assessed by analysing Hardy-Weinberg equilibrium (HWE) and missing genotype data. Only a small proportion of the markers with HWE deviations (4.08%) and markers with missing genotypes above 0.10 (2.28%) are included in CNV regions.

## Discussion

This work has generated over 200 million genotypes, the largest study of this kind in Spain. Detailed information of the genetic structure of the Spanish population will serve as a reference framework for future GWAS studies in Spain, and will be shared with other researchers via external National Public Health evaluation and approval.

We have characterized the genetic structure of the population of Spain, describing genome-wide LD patterns, haplotype blocks, population structure and copy-number variants in a sample of over 800 unrelated Spanish individuals. The individuals that participated in the study were recruited by a random sampling approach from a cross-sectional population-based epidemiological survey from eight locations in Spain, representing different geographical locations across the country (South, Central, North-East and North-West). The recruiting centers include both small rural clinics as well as large hospitals close to major metropolitan areas. Individuals that reported a different nationality were not included in the study. Therefore, the sample can be considered as representative of the general Spanish population.

These samples were genotyped at Neocodex with an Affymetrix Nsp I 250 K chip. The high call-rate (99.1%) speaks of the high quality of the genotyping performed. Although there are now commercial genotyping chips that provide a more complete coverage of the genome, at the starting point of this project this Nsp I 250 K chip was the best possible choice, and provides enough genotype information for the current project.

### LD and haplotypic structure

A major finding of the present study is that the Spanish population is generally similar to the CEU HapMap sample (of Northern and Western Europe origin), but also largely homogeneous within itself. Numerous pieces of evidence point to this conclusion. For example, a significant proportion of the SNPs analyzed were monomorphic (2.3%) or rare (10.2%), even in this large sample of 801 individuals. In comparison, the CEU dataset yielded 15.1% of monomorphic or rare SNPs, but in a much smaller sample of only 60 individuals (over 13 times smaller). This large amount of SNPs with no or very little variability is a sign of the homogeneity of the Spanish population.

The LD patterns observed in this sample of the Spanish population is similar to the patterns observed in the CEU HapMap sample. This is not surprising since the level of genetic differentiation within Europe is small [7]. We detect LD extending over large distances in the Spanish population, but less than in the CEU sample. We have also found a large number (33,037) of haplotype blocks. These blocks are generally closely located to the blocks detected in the CEU sample, but in the Spanish sample there are more blocks, and smaller on average. These findings could be an artefact due to the difference in sample size between the two samples, but may indeed be reflecting the more complex origin of the current Spanish population [9]. Indeed, these results confirm the suggestion that the Spanish population has more haplotypic diversity than Northern/Western Europeans [9]. This is a possible scenario, given that the Iberian Peninsula has been under large and long-lasting migratory influences, and admixture, from other European, Mediterranean, and North African populations.

Another interesting finding is that a larger portion of the genome is covered by blocks in the Spanish sample (28%), than in the CEU sample (24%). This finding is again probably due to the larger Spanish sample, so that the 1602 chromosomes analyzed probably revealed more rare haplotypes, therefore enlarging the proportion of the chromosome covered by haplotype blocks. This extra block coverage in the Spanish sample may turn useful for association studies, although this is probably a characteristic of other large homogeneous samples.

These results suggest that the general Spanish population, as characterized in the present study by sampling from eight different cities widely-spaced across Spain, is generally similar to other European populations, although more genetically diverse than Western and Northern Europeans. Moreover, the Spanish population is remarkably homogeneous within itself in terms of global genetic structure. In view of these results, the population of Spain is sufficiently genetically similar to the CEU sample so that the CEU HapMap dataset could be used to infer genotypes for the Spanish population. Nonetheless, in spite of their general similarity, there are substantial differences between these two European subgroups, and therefore imputed data from the HapMap study many not describe some particular genetic patterns of the Spanish population. The dataset in this study can be extremely useful to compare allele and haplotype frequencies against the CEU sample, and to estimate the confidence of imputed genotypes in all regions of the genome. It is important to note here that some of the differences found between the Spanish and the CEU samples may be due to the difference in size among both samples.

### Population stratification

The results of our population structure analyses are consistent with no major population stratification present in this sample of the Spanish population. This result is reassuring since individuals reporting nationalities other than Spanish were excluded from the study. Both, Structure and PC results with a set of 2,050 uncorrelated SNPs showed no evidence of genetic diversity in the sample.

In addition, we were able to analyze fine structure within this sample by running PC analysis using a large set of markers (102,850 SNPs). The results of this second analysis are also consistent with prior reports that were able to predict locations of origin within a 700 Km radius using different European populations [5,7], and other studies that found subtle differences between locations within a country [10,11]. In our sample, following a similar strategy, we were able to differentiate between the two more geographically distant centers. Furthermore, these observed differences seem to correspond to the same geographical axis that has been previously found in European populations. This fine structure can be the result of genomic regions that show strong geographic variation [16] and may be more evident in small, rural or isolated samples than in major cities where subpopulations tend to mix [17]. This potential source of bias should be taken into account in association studies. It is worth noting that our sample of the Spanish population was quite homogeneous, and the genomic inflation factor (based on median chi-squared), as estimated by the software Plink [18], was exactly 1, as expected when only one population is being analyzed, but still specific genomic regions need to be carefully reviewed.

### CNV

We have also defined the first CNV map in the Spanish general population. According to our data, 2.35% of the human genome (autosomes and chromosome X) is susceptible to structural variants. This estimation is in range with previously published studies analysing structural variants with the Affymetrix platform [19-21].

We detected a wide range of CNVs population frequencies, although only 34.35% of these variants had a population frequency above 1%. 301 of the CNVs described in this work are fully covered by previously described structural variants. In addition, another ten CNVs have 90% or more of their nucleotides represented in previous CNVs. These 311 CNVs are therefore supported by at least one independent study. The remaining 312 CNVs are also included in Additional File 2 but for descriptive purposes only. These CNVs need to be confirmed in independent datasets. Indeed, because we have analyzed 799 samples, some of these CNVs could be low frequency or population specific variants which went undetected in previous studies with smaller sample sets.

We have confirmed in this study that nonallelic homologous recombination (NHR) could explain the origin of about 33% of CNVs. Interestingly, those CNVs are more frequent than other variants out of rearrangement hotspot regions and they represent 46.50% of all CNVs detected in this study. Regardless of the frequency of NHR events, we estimate that a considerable proportion of CNVs in the normal population may be a consequence of NHRs.

Most of the CNVs detected in our study overlap with known genes, and of those, 157 CNVs (25.22%) overlap with 125 disease loci. This observation is in agreement with previous results. For instance, the 38,406 structural variant regions included in DGV overlap 1183 disease loci. There exist several plausible reasons for these observations, such as the existence of false positives in CNV genome-wide surveys, inaccurate disease-frequency estimates, embryonic lethality effect for homozygous deletions of specific genes, misclassification of samples as normal controls, and rescue of the altered gene function by other related gene product [22].

In our sample set, only 43 (6.90%) CNVs overlapping disease loci have a population frequency above 1%, and none of them include homozygous deletions. From those, only three CNVs exceed 10% in population frequency and all of them are completely covered by previously described structural variants. Two of these CNVs are contiguous on chromosomal region 15q11.2, one of the most unstable regions in the human genome [23]. These two CNVs overlap the genes *hect domain and RLD 2 *(*HERC2*) associated with skin, hair and eye pigmentation (OMIM: 227220), and *BCL8 B-cell CLL/lymphoma 8 *(*BCL8*) which has been implicated potentially in B-cell lymphoma (OMIM: 601889). The third CNV is located at 19p13.13 and overlaps with the gene *RNASEH2A ribonuclease H2, subunit A *(*RNASEH2A*) whose mutations may be responsible for the Aicardi-Goutieres syndrome (OMIM: 610333). Interestingly, this is a severe autosomal recessive disorder that mimics *in utero *viral infections and therefore its real incidence could be underestimated [24].

All these data suggest that some disease loci could be located within genomic regions that are prone to structural alterations. This observation has potential implications on the molecular diagnosis and on the disease frequency estimations of the phenotypes.

Finally, our results suggest that structural variants could be responsible for a small percentage of the Hardy-Weinberg deviations and missing genotypes commonly observed in genome-wide surveys. Therefore, it is advisable to consider the existence of such structural variants for specific SNPs when Genome Wide Association Studies are (GWAS) performed.

## Conclusions

In summary, we have performed a deep characterization of our reference control population for GWAS and confirmed that the Spanish population is sufficiently homogeneous to conduct genetic association studies with minor risk of population stratification. In addition, the results obtained, together with other concomitant efforts underway in other European countries, will be useful to shed light on the nature of European genetic diversity and the Spanish population genomic history. Complete data and further details of our study, including raw genotypes, can be accessed after external Ethical Committee review and Public administrative authorisation.

## Methods

### Sample

The dataset includes 825 unrelated individuals recruited by a random sampling approach from a cross-sectional population-based epidemiological survey performed in eight different cities of Spain, including Alicante, Arévalo (Ávila), Avilés (Asturias), Málaga, Mérida (Badajoz), Segovia, Talavera (Toledo), and Vic (Barcelona). The recruiting centers include both small rural clinics as well as large hospitals close to major metropolitan areas from across the country (South, Central, North-East and North-West). Individuals that reported a different nationality were not included in the study. Therefore, the sample can be considered as representative of the general Spanish population. The goal of the survey was to investigate the prevalence in the Spanish population of anthropometric and physiological parameters related to obesity and other components of the metabolic syndrome [25,26]. The sample includes a total of 450 males (54.5%), and 375 females (45.5%), with an average age of 52 (SD = 8.84) years old, and a range 34-76.

Identity-By-State (IBS) sharing can identify sample duplications or related individuals. Genome-wide IBS estimates suggested the presence of 19 pairs of siblings, two sibling trios, and one parent-offspring pair, and therefore 24 individuals were removed to eliminate these relationships. The remaining samples (N = 801) used in this study grouped together in a broad cluster of diverse ranges of relatedness. All study subjects gave their written informed consent to participate in the study. The study protocol was approved by the Ethics Committee of the Hospital Clínico San Carlos of Madrid.

In addition, for the LD and haplotype analysis, 60 unrelated individuals from the CEU HapMap dataset were also used for comparison with the Spanish sample [1]. The CEU dataset is composed of Utah residents with ancestry from northern and western Europe, and whose samples were collected by CEPH in 1980. For these individuals, we selected only the same SNPs that were genotyped in the Spanish sample. Both datasets were applied the same quality control process. Moreover, datasets from HapMap phase 3 release 3 http://hapmap.ncbi.nlm.nih.gov/ were also employed in the Principal Components analysis. More precisely we used Hap Map datasets of unrelated individuals with European (CEU: Utah residents with ancestry from northern and western Europe, and TSI: Toscani in Italy), African (ASW: African ancestry from Southwest USA, LWK: Luhya in Webuye, Kenya; MKK: Maasai in Kinyawa, Kenya; YRI: Yoruba in Ibadan, Nigeria) and Asian ancestry (CHB: Han Chinese in Beijing, China; CHD: Chinese in Metropolitan Denver, Colorado; GIH: Gujarati Indians in Houston, Texas; JPT Japanese in Tokyo, Japan) [27].

### DNA extraction

DNA extraction from frozen peripheral blood was performed in a MagNa Pure LC Instrument (Roche Diagnostics), using MagNa Pure LC DNA Isolation Kit (Roche Diagnostics) in accordance with the manufacturer's instructions.

### Genotyping and Quality Control

All samples were genotyped using the Affymetrix Nsp I 250K chip, that includes 262,264 SNP markers (256,512 on autosomes, 5705 on sex chromosomes, and 47 control markers).

This chip provides a good coverage of the genome with an average SNP density of 1 SNP every 11 kb (median 1 SNP per 5 kb), and an average heterozygosity of 0.3. Genotypes were read and called with standard Affymetrix software (GCOS, GTYPE, Genotyping Console, BRLMM) using default parameters, and exported as linkage-format files.

All SNPs in the autosomal chromosomes were subjected to quality control filters, specifically a minor allele frequency (MAF) equal or larger than 10%, a SNP call-rate equal or larger than 90%, and a p-value for Hardy-Weinberg equilibrium (HWE) larger than 10xE-4. Regarding the minor allele frequency, 2.3% of the SNPs were monomorphic, 10.2% were rare alleles (MAF = 0-1%), and 20.4% were low-frequency alleles (MAF = 1-10%). Moreover, all samples yielded a call rate above 93%, as required by the BRLMM software. The average sample call rate was 99.1%, with a range 93.9-99.8%. In addition, the average SNP call rate was 99.1%, with a range 68.2-100%. 0.8% of the SNPs had call-rates below 90%, and 2.6% had call-rates between 90-95%. Finally, for our sample of 801 individuals we decided, based on simulations and Q-Q plots, that 10xE-4 was a sensible HWE cut-off value. We found that 2.0% of the SNPs had a p-value for the HWE test lower than 10xE-4.

In summary, 67.0% of the SNPs had a MAF = > 10%, 99.2% had call-rates above 90%, and 98% passed the HWE test. Overall, 64.9% (166,588) of all autosomal SNPs passed our quality control.

### Analysis

Plink [18] was employed to manage the datasets and perform quality control filters such as call rate, MAF, HWE, and Identity-By-State (IBS) estimates. GRR [28] was also employed to estimate IBS and visualize the resulting relationships.

LD and haplotype blocks were estimated with Haploview [29]. Pair-wise LD was measured with Lewontin's standardized deviation coefficient (D') and with pair-wise correlation coefficient (r2). Haplotypes were estimated using the Gabriel definition [30] with all the defaults parameters as implemented in Haploview. LD and haplotype blocks were analyzed for each chromosome separately. Moreover, due to computer (RAM) limitations, each arm of the first six chromosomes was analyzed independently.

We explored the presence of population stratification in our study sample by using two different available software: STRUCTURE and EIGENSOFT. In order to run STRUCTURE a small subset of unlinked markers were selected using Plink, by excluding all SNPs with a pair-wise genotypic r^2 ^greater than 1.1% with sliding windows of 200 SNPs (with increments of 5 SNPs between windows). A total of 2,050 SNPs (subset A) from the 166,559 that passed the quality control were identified. STRUCTURE uses a model-based clustering method for analyzing multilocus genotype data to infer population structure and assign individuals to populations [31]. We tested different scenarios assuming a different number of underlying populations (k equals to 1 through 4) allowing a large number of iterations (25 K in the burn-in period followed by 500 K repetitions). We estimated the mean log likelihood of the data for a given k (referred to as L(K)) in each run. Furthermore we performed multiple runs for each value of k computing the overall mean L(K) and its standard deviation.

Additionally we run a Principal Components (PC) Analysis using EIGENSOFT *smartpca *command [32]. We determined the principal components using two different subsets of SNPs. The first subset (subset A) corresponds to the same 2050 SNPs from the STRUCTURE analyses. Since PC Analysis, unlike STRUCTURE, can handle a large number of SNPs we performed a second analysis in which less stringent SNP selection was performed following a two-tier strategy analogous to one previously described in the literature [7]. This approach has been successfully used to detect fine structure of European populations, being able to predict the reported origin in 90% of cases within a 700 Km distance [7]. Briefly, we first used Plink to exclude all SNPs with a pair-wise genotypic r^2 ^greater than 80% with sliding windows of 50 SNPs (with increments of 5 SNPs between windows). In a second step, in order to exclude chromosomal regions showing long-range LD we ran a preliminary PC Analysis estimating the weights of each individual SNP in each one of the top 6 PCs. For each PC we excluded those regions of up to 4 Mb of length with either more than 2 SNPs among the top 10 contributing SNPs or 5 SNPs among the top 100 contributing SNPs. This resulted in excluding several regions, such as regions of known long-range LD like the Major Histocompatibility Complex (MHC) region (Table 2). This entire process resulted in a second subset (subset B) that consisted of a total of 102,850 SNPs. For both subsets A and B we run PC Analyses to obtain the top PCs. We run two additional PC Analyses in which Hap Map datasets were included (European datasets only in a first instance and then Hap Map datasets from European, African and Asian ancestry). These additional anayses were performed using markers in subset B only. We analyzed population stratification graphically by plotting all individuals according to the top two PCs in each analysis. In order to evaluate statistical significance of PCs, Tracy-Wisdom tests PC were also carried out.

**Table 2:** Long-range LD regions across the genome. These regions were excluded from subset B for the PC analysis.

Chromosome	Long-range LD Region
3	52.5-56.5 Mb
4	70.5-76.5 Mb
6	26.0-32.0 Mb
6	124.0-128.0 Mb
8	8.0-12.0 Mb
8	12.5-16.5 Mb
12	38.5-42.5 Mb
14	45.5-49.5
21	27.5-31.5
21	127.5-131.5 Mb

Copy Number Variant (CNV) analyses were carried out in the full SNP set by using the Copy Number Analysis Tool (CNAT) v.4.0 software (Affymetrix, Santa Clara) following the manufacturer's instructions. We selected 25 control female samples from other ongoing projects as the reference group. All the samples in this study (n = 801) passed the IQR quality control with the exception of two samples that were removed from further CNV analyses. Overall, our CNV sample set is composed of 799 samples. Statistical analyses were carried out using Statistical Package for Social Sciences (SPSS) software v.13.0.

As online resources, we used the hg18.knownGene and hg18.refGene tables to build a gene map interval in the autosomes and chromosome X. The table hg18.dgv was used to retrieve information about structural variants from the Database of Genomic Variants (DGV, http://projects.tcag.ca/variation/) and the table hg18.genomicSuperDups to define rearrangement hotspot regions. All these tables were downloaded from the Table Browser at the UCSC Genome Bioinformatics resource http://genome.ucsc.edu/. Galaxy browser tools were used to manage genomic intervals [33]http://main.g2.bx.psu.edu/. Information about OMIM genes and phenotypes were extracted from mim2gene.txt and morbidmap.txt tables at NCBI FTP site ftp://ftp.ncbi.nlm.nih.gov/repository/OMIM/.

## Authors' contributions

JG, JJG, AG-P, and MES participated in the design of the study, analyzed and interpreted the data, and drafted the manuscript. MTM-L and CZ acquired and managed the clinical data into the database. MCR, AS, RR-L, FJM, JLR, CM-R, JV, and JMC managed Neocodex's biobank, performed DNA extraction, DNA genotyping and appropriate quality controls during this project. EM, CO, MDO, MG, MR, RP, AR-A, and JLS-G managed Neocodex's clinical database and provided administrative, technical and computer support. MS-R was the called Director of the VIVA Study and Principal Investigator of the Segovia Survey, and intervened in the research design of these population-based studies, including recruitment strategies, selection of anthropometric measurements and biochemical phenotyping, and DNA banking. EV, LMR and AR designed Neocodex's biobank and its associated database, conceived of this study, and obtained the funds to execute it. All authors read and approved the final manuscript.

## Competing interests

At the time this research was conducted, JG, JJG, AG-P, MES, MCR, AS, RR-L, FJM, JLR, CM-R, JV, JMC, EM, CO, MDO, MG, MR, RP, AR-A, JLS-G, EV, LMR, and AR were all employees and/or shareholders of Neocodex.

## Supplementary Material

Additional file 1 **List of haplotype blocks across all autosomal chromosomes in the Spanish population**. Each block is described by the SNPs that compose it, the major haplotypes with haplotypic frequencies, and the LD (D') among adjacent blocks. For each chromosome, the correspondence list between the SNP names and numbers is included in a separate table. Click here for file

Additional file 2 **List of the 623 CNVs identified in this study**. For each CNV, this table includes the physical positions, the copy number states (CN_state) coded as 0: copy number losses, 2: copy number gains and losses, and 4: copy number gains. The column 'individuals' shows the number of individuals in our sample set with the corresponding CNV. The column 'coverage' indicates the percentage of the CNV covered by previously described structural variants at DGV database. Click here for file
